# Effect of Prehospital Time on Door‐to‐Computed Tomography Time in Acute Ischemic Stroke: A Multicenter Study

**DOI:** 10.1002/brb3.71656

**Published:** 2026-08-05

**Authors:** Kuo‐En Chen, Po‐Chun Wang, Sheng‐Feng Lin, Hui‐An Lin

**Affiliations:** ^1^ School of Medicine, College of Medicine Taipei Medical University Taipei Taiwan; ^2^ Department of Public Health, School of Medicine, College of Medicine Taipei Medical University Taipei Taiwan; ^3^ School of Public Health, College of Public Health Taipei Medical University Taipei Taiwan; ^4^ Center of Evidence‐Based Medicine Taipei Medical University Hospital Taipei Taiwan; ^5^ Department of Medical Research Taipei Medical University Hospital Taipei Taiwan; ^6^ Department of Emergency Medicine Taipei Medical University Hospital Taipei Taiwan; ^7^ Department of Emergency Medicine, School of Medicine, College of Medicine Taipei Medical University Taipei Taiwan

**Keywords:** computed tomography (CT), door‐to‐CT time, emergency department, emergency medical services (EMS), ischemic stroke

## Abstract

**Objectives:**

Timely brain imaging is critical for optimizing stroke treatment outcomes. Delays at the prehospital stage significantly impact acute ischemic stroke management. Computed tomography (CT) plays a vital role in guiding treatment decisions.

**Methods:**

We conducted a retrospective observational study using data from the Acute Stroke Statistics System of Taipei City, covering the period from January 1, 2011, to June 30, 2022. The association between prehospital time intervals and door‐to‐CT time was analyzed in patients with acute ischemic stroke. Logistic regression models were applied, with door‐to‐CT time <25 min as the dependent variable. Independent variables included patient characteristics, comorbidities, and prehospital time components: decision‐making time (symptom onset to emergency medical service [EMS] activation), EMS response time, on‐scene time, and transport time.

**Results:**

Among 2351 acute ischemic stroke patients, 1734 patients with complete documentation were included in the final analysis. Factors associated with a door‐to‐CT time >25 min included age ≥85 years (OR: 1.387; 95% CI: 1.025–1.878; *p* = 0.034), hypertension (OR: 1.412; 95% CI: 1.047–1.905; *p* <0.001), decision‐making delay (OR: 1.001; 95% CI: 1.001–1.002; *p* <0.001), and decision‐making delay ≥160 min (OR: 2.340; 95% CI: 1.766–3.099; *p* <0.001). Prehospital notification was associated with a lower likelihood of delayed imaging (OR: 0.371; 95% CI: 0.266–0.517; *p* < 0.001). In multivariable analysis, decision‐making delay ≥160 min (OR: 2.279; 95% CI: 1.711–3.036; *p* <0.001) remained significant determinants of delayed CT imaging, whereas prehospital notification (OR: 0.382; 95% CI: 0.272–0.536; *p* <0.001) served as a significant protective factor against delayed imaging. A two‐stage pooled analysis across nine advanced emergency responsibility hospitals in Taipei City confirmed these findings with low heterogeneity.

**Conclusions:**

Decision‐making delays and prehospital notification appear to be key determinants of door‐to‐CT time in the management of acute ischemic stroke.

## Graphical abstract

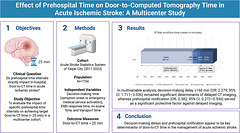

## 1 Introduction

Stroke imposes a substantial long‐term disease burden on the global population (GBD 2019 Stroke Collaborators 2021), with nearly half of all stroke survivors experiencing functional impairment (Donkor 2018; Katan and Luft 2018). The standardized disability‐adjusted life‐years attributable to stroke exceed 140 million worldwide (GBD 2019 Stroke Collaborators 2021). Acute ischemic stroke, the most common type of stroke, accounts for approximately 70%–80% of all cases (Benjamin et al. 2019; Murray et al. 2012). The concept of “time is brain” underscores the urgent need for rapid diagnosis and intervention in acute ischemic stroke (Saver 2006). Notably, for each minute of cerebral ischemia, approximately 1.9 million neurons, 14 billion synapses, and 12 kilometers of myelinated fibers are lost due to reduced blood flow and oxygen deprivation (Saver 2006; Butcher et al. 2003; Gomez 2018; Heiss 2011). This underscores the importance of timely revascularization to preserve neurological function and mitigate long‐term disability. Current guidelines established by the American Heart Association (AHA) recommend intravenous thrombolysis (IVT) within 4.5 h and intra‐arterial thrombectomy (IAT) within 24 h of symptom onset for patients selected on the basis of brain imaging (Powers et al. 2018). Delays beyond these therapeutic windows markedly diminish the benefits of revascularization and increase the risk of complications, including symptomatic intracranial hemorrhage (Saver et al. 2016). For eligible patients, IVT should be administered within 60 min of arrival at the emergency department (ED) because each 15‐min reduction in door‐to‐needle time is associated with a 5% reduction in the risk of in‐hospital mortality (Jauch et al. 2013).

Diagnostic imaging, particularly computed tomography (CT) and CT angiography, is essential for guiding treatment decisions in acute ischemic stroke. Before IVT, noncontrast brain CT must be performed to rule out intracranial hemorrhage. The AHA recommends a door‐to‐CT time of <25 min for prompt decision‐making (Adams et al. 2007). Rapid imaging is crucial for determining the feasibility of recanalization therapies. A clinical trial (2024) demonstrated the efficacy of tenecteplase in treating patients with acute ischemic stroke between 4.5 to 24 h after symptom onset when perfusion imaging or angiographic evidence supported intervention (Albers et al. 2024). These findings emphasize the pivotal role of timely diagnostic imaging in identifying eligible candidates for thrombolysis and ensuring prompt treatment.

Given the pivotal role of timely CT imaging in the management of acute ischemic stroke, delays occurring at both the prehospital and in‐hospital stages can critically undermine treatment outcomes. Prehospital delays, reported to range from 0.8 to 24 h with median times clustering around 3 to 4 h, remain the primary bottleneck in the stroke care pathway (Evenson et al. 2009). These prehospital delays are influenced by multiple factors, including patient decision‐making, geographic accessibility, and the availability and efficiency of emergency medical services (EMS) (Faiz et al. 2014; Cash et al. 2022). In contrast, in‐hospital delays often stem from delayed stroke recognition, high clinical workloads, and the complexity of managing concurrent medical conditions, all contributing to prolonged door‐to‐CT intervals (Centers for Disease Control and Prevention (CDC) 2007). Across Asia, Europe, and North America, 19 studies have documented median door‐to‐CT times spanning from 0.1 to 3.1 h, reflecting considerable heterogeneity influenced by socioeconomic conditions, healthcare infrastructure, and emergency response systems (Evenson et al. 2009; Dhand et al. 2024). While both prehospital and in‐hospital delays are recognized as critical determinants of treatment latency, few investigations have directly examined how prehospital time intervals shape subsequent in‐hospital processes, particularly door‐to‐CT time. To bridge this knowledge gap, our multicenter study seeks to evaluate the impact of prehospital delays on in‐hospital imaging timelines among patients with acute ischemic stroke in Taipei, the capital city of Taiwan.

## 2 Methods

### 2.1 Study Design and Population

This retrospective observational study analyzed the effect of prehospital time on the timeliness of brain CT imaging in patients with acute ischemic stroke. The study protocol was approved by the Joint Institutional Review Board of Taipei Medical University, Taiwan (approval number: N202110064). The requirement for informed consent was waived because all data used were anonymized and deidentified. The study population was derived from the Acute Stroke Statistics System of Taipei City dataset, managed by the Taipei City Fire Department. The dataset comprises records collected between January 1, 2011, and June 30, 2022. Patient data were collected by emergency medical technicians (EMTs) at the scene and by emergency physicians and neurologists during comprehensive evaluation and treatment at nine advanced emergency responsibility hospitals in Taipei City (Table S1). In Taiwan, advanced emergency responsibility hospitals manage critical medical situations. For patients with acute ischemic stroke, these hospitals provide IVT and arterial thrombectomy on a 24/7 basis. Patients with incomplete clinical records were excluded from the analysis.

### 2.2 Data Collection

Demographic variables assessed in this study included sex and age, whereas documented past medical history included hypertension, diabetes mellitus, dyslipidemia, and atrial fibrillation. The prehospital course was categorized into four time intervals (Schwartz et al. 2016; Acker et al. 2007; Zhang et al. 2023): decision‐making time, the time from symptom onset to EMS activation; EMS response time, the time from EMS dispatch to EMT arrival at the scene; on‐scene time, the time EMTs spent at the scene evaluating and stabilizing the patient; and transport time, the time from departure from the scene to hospital arrival. Door‐to‐CT time was defined as the interval from ED triage to the completion of brain CT. The severity of ischemic stroke was assessed upon ED arrival by using the National Institute of Health Stroke Scale. Revascularization therapy was categorized as follows: IVT alone, IAT alone, and IAT following IVT.

### 2.3 Outcome Measures

We evaluated the effect of prehospital time on door‐to‐CT time, focusing on the association between prehospital time and a door‐to‐CT time of >25 min. Additionally, factors associated with prolonged door‐to‐CT time (>25 min) were analyzed to identify potential delays in acute ischemic stroke management.

### 2.4 Statistical Analysis

Descriptive statistics were used to summarize data. Categorical variables are presented in terms of number and percentage values, whereas continuous variables are presented in terms of mean and standard deviation values. Between‐group comparisons of categorical variables were performed using the chi‐square test or Fisher exact test, whereas between‐group comparisons of continuous variables were performed using the Student's *t*‐test or Mann–Whitney *U* test. Age was analyzed as both continuous and categorical variables. Patients were stratified into four groups by age: <55, 55–69, 70–84, and ≥85 years (Li et al. 2021). Logistic regression was performed to evaluate the odds ratios (ORs) for the effect of prehospital time on door‐to‐CT time. The regression model was structured as follows: log (*y*/1 − *y*) = *β*_0_ + *β*_1_ *X*_1_ + *β*_2_ *X*_2_, where *y* represents patients with a door‐to‐CT time of >25 min, 1 − *y* represents patients with a door‐to‐CT time of ≤25 min, *β*_0_ represents the intercept, *X*_1_ represents prehospital time with *β*_1_ as corresponding OR, and *X*_2_ represents a confounding factor with *β*_2_ as the corresponding OR. Subgroup analyses for the nine hospitals were performed using multivariate logistic regression models to calculate ORs. In cases where dispersion was observed in the multivariate models, exact logistic regression models were applied to calculate the ORs. A two‐stage pooled analysis was performed, in which hospital‐specific effect estimates were first derived and subsequently combined using a DerSimonian–Laird random‐effects model. Heterogeneity across hospitals was assessed using the Higgins *I* ^2^ statistic, with an *I* ^2^ value of >50% indicating substantial heterogeneity. Potential biases were evaluated using funnel plots, Galbraith plots, and Egger's and Begg's tests. Statistical analyses were performed using SPSS (version 22.0; IBM Corporation, Armonk, NY, USA) and Stata (version 17; StataCorp, College Station, TX, USA). A two‐sided *p* value of <0.05 was considered to be statistically significant.

## 3 Results

### 3.1 Population Characteristics

**FIGURE 1:**
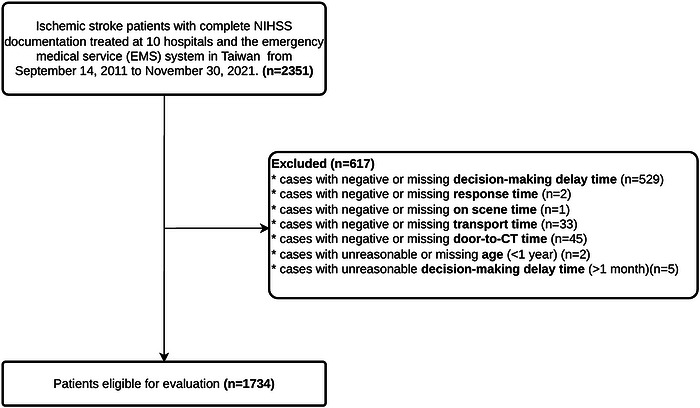
Flowchart depicting the selection of eligible acute ischemic stroke patients from the Acute Stroke Statistics System of Taipei City dataset (2011–2022). CT, computed tomography; EMS, emergency medical services; NIHSS, National Institutes of Health Stroke Scale.

During the study period, a total of 2351 records were available in the registry. Exclusions were made for records with negative or missing values for decision‐making delay (529 records), EMS response time (2 records), on‐scene time (1 record), transport time (33 records), and door‐to‐CT time (45 records). After these exclusions, 1741 cases remained. Furthermore, cases with unreasonable or missing age values (e.g., age <1 year; 2 records) were excluded, resulting in 1739 valid records. Finally, five cases with implausible decision‐making delays exceeding 1 month were eliminated, yielding a final cohort of 1734 patients with acute ischemic stroke. A flowchart depicting patient selection is presented in Figure 1.

Among the included patients, 1490 (85.9%) underwent brain CT within the recommended door‐to‐CT time of ≤25 min, whereas 244 patients (14.1%) had a door‐to‐CT time of >25 min. The patients’ clinicodemographic characteristics are summarized in Table 1. Patients with a door‐to‐CT time of >25 min were significantly older than those with a door‐to‐CT time of ≤25 min (mean age: 75.9 ± 12.0 vs. 73.7 ± 13.3 years; *p* = 0.016). The proportion of patients aged ≥85 years was higher in the >25 min group than in the ≤25 min group (28.7% vs. 22.5%; *p* = 0.034). A prior diagnosis of hypertension was more prevalent in the >25 min group than in the ≤25 min group (72.1% vs. 64.7%, *p* = 0.023). Furthermore, the rate of EMS prenotification differed significantly between the >25 and ≤25 min groups (75.0% vs. 89.0%; *p* < 0.001).

**TABLE 1:** Clinicodemographic characteristics of the study population (*n* = 1734).

Characteristics	Door‐to‐CT time ≤ 25 min (*n* = 1490 [85.9%])	Door‐to‐CT time > 25 min (*n* = 244 [14.1%])	*p*
Sex (male)	845/1490 (56.7%)	136/244 (55.7%)	0.776
Age (years)	73.71 ± 13.34	75.90 ± 12.03	0.016*
<55	127/1490 (8.50%)	11/244 (4.50%)	0.032*
55–69	399/1490 (26.8%)	65/244 (26.6%)	0.964
70–84	555/1490 (37.2%)	85/244 (34.8%)	0.469
≥85	335/1490 (22.5%)	70/244 (28.7%)	0.034*
Hypertension	964/1490 (64.7%)	176/244 (72.1%)	0.023*
Diabetes mellitus	401/1490 (26.9%)	74/244 (30.3%)	0.268
Atrial fibrillation	389/1490 (26.1%)	56/244 (23.0%)	0.295
Dyslipidemia	377/1490 (25.3%)	71/244 (29.1%)	0.209
Prenotification	1326/1490 (89.0%)	183/244 (75.0%)	<0.001*
National Institute of Health Stroke Scale at the emergency department	11.42 ± 8.06	10.56 ± 9.17	0.129
Decision‐making delay	129.56 ± 275.88	350.78 ± 801.20	<0.001*
EMS response time	4.31 ± 2.14	4.27 ± 2.03	0.812
On‐scene time	13.52 ± 5.40	13.78 ± 4.80	0.490
Transport time	9.47 ± 4.79	10.07 ± 4.09	0.065
Total prehospital time	156.86 ± 276.42	378.90 ± 801.03	<0.001*

*Note*: Categorical variables are presented as numbers (percentages) and compared using the chi‐square test or Fisher's exact test. Continuous variables are presented as mean ± standard deviation and compared using the Student's *t*‐test or Mann–Whitney *U* test. *p* < 0.05 indicates statistical significance (marked with *).

CT, computed tomography; EMS, emergency medical service.

### 3.2 Prehospital Time Intervals

**FIGURE 2:**
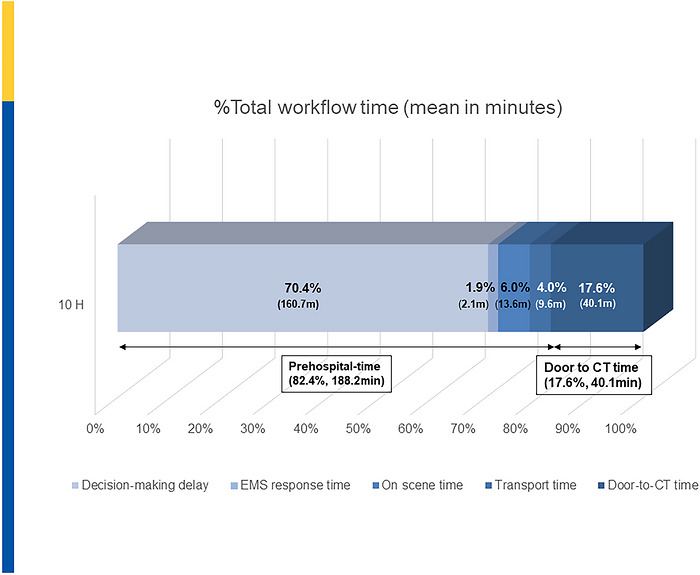
Timeline from the onset of symptoms to the completion of computed tomography. Time intervals are presented as mean values (minutes) and as a percentage of the total overall workflow duration. CT, computed tomography.

Compared with patients with a door‐to‐CT time of ≤25 min, those with a door‐to‐CT time of >25 min exhibited significantly long decision‐making delay (129.6 ± 275.9 vs. 350.8 ± 801.2 min; *p* < 0.001) and total prehospital time (156.9 ± 276.4 vs. 378.9 ± 801.0 min; *p* < 0.001). However, no significant between‐group difference was noted in EMS response time, on‐scene time, or transport time. The independent contribution of each prehospital time interval toward door‐to‐CT time in the overall stroke care timeline is presented in Figure 2. The simple logistic regression analysis of prehospital workflow (Table 2) revealed that both decision‐making delay (OR: 1.001; 95% confidence interval [CI]: 1.001–1.002; *p* < 0.001) and prolonged total prehospital time (OR: 1.001; 95% CI: 1.001–1.002; *p* < 0.001) were significantly associated with an increased likelihood of door‐to‐CT time exceeding 25 min.

**TABLE 2:** Association between prehospital time and a door‐to‐CT time of >25 min, univariate analysis of data from nine advanced emergency responsibility hospitals (2011–2022).

(Unit: min)	Door‐to‐CT time > 25	*p*
Decision‐making delay	1.001 (1.001–1.002)	<0.001*
EMS response time	0.992 (0.930–1.058)	0.812
On‐scene time	1.009 (0.984–1.034)	0.490
Transport time	1.023(0.997–1.048)	0.078
Total prehospital time	1.001(1.001–1.002)	<0.001*

*Note*: Data are presented as odds ratios (ORs) with 95% confidence intervals (CIs). *p* < 0.05 indicates statistical significance (marked with *).

CT, computed tomography.

### 3.3 Factors Associated With Door‐to‐CT Time

Key factors associated with door‐to‐CT time are presented in Table 3. In the univariate analysis, the following factors were significantly associated with a door‐to‐CT time of >25 min: age (OR: 1.013; 95% CI: 1.002–1.024; *p* < 0.001), age ≥ 85 years (OR: 1.387; 95% CI: 1.025–1.878; *p* = 0.034), prehospital notification (OR: 0.371; 95% CI: 0.266–0.517; *p* < 0.001), hypertension (OR: 1.412; 95% CI: 1.047–1.905; *p* < 0.001), decision‐making delay (OR: 1.001; 95% CI: 1.001–1.002; *p* <0.001), and decision‐making delay ≥ average time (160 min; OR: 2.340; 95% CI: 1.766–3.099; *p* < 0.001).

**TABLE 3:** Factors associated with prolonged door‐to‐computed tomography time (>25 min) in patients, univariate and multivariate analyses.

	Univariate analysis		Multivariate analysis model 1		Multivariate analysis model 2	
	OR (95% CI)	*p*	OR (95% CI)	*p*	OR (95% CI)	*p*
Age (years)	1.013 (1.002–1.024)	0.016*	1.009 (0.998–1.020)	0.114		
Age ≥ 85 years	1.387 (1.025–1.878)	0.034*			1.230 (0.900–1.681)	0.195
Prenotification	0.371 (0.266–0.517)	<0.001*	0.381 (0.271–0.534)	<0.001*	0.382 (0.272–0.536)	<0.001*
Hypertension	1.412 (1.047–1.905)	0.024*	1.254 (0.921–1.708)	0.151	1.288 (0.948–1.750)	0.105
Decision‐making delay	1.001 (1.001–1.002)	<0.001*				
Decision‐making delay ≥ average (160 min)	2.340 (1.766–3.099)	<0.001*	2.273 (1.707–3.027)	<0.001*	2.279 (1.711–3.036)	<0.001*

*Note*: Model 1 is adjusted for age as a continuous variable; Model 2 is adjusted for age as a categorical variable. Data are presented as odds ratios (ORs) with 95% confidence intervals (CIs). *p* < 0.05 indicates statistical significance (marked with *).

Multivariate logistic regression models were adjusted for age as a continuous (Model 1) or categorical variable (Model 2). In both models, prenotification remained a significant protective factor against a door‐to‐CT time of >25 min (Model 1 vs. Model 2, OR: 0.381 [95% CI: 0.271–0.534; *p* < 0.001] vs. 0.382 [95% CI: 0.272–0.536; *p* < 0.001]), reinforcing its role as a robust independent predictor of timely imaging. Conversely, a decision‐making delay of ≥ 160 min remained a significant independent predictor of delayed CT imaging (Model 1 vs. Model 2, OR: 2.273 [95% CI: 1.707–3.027; *p* < 0.001] vs. 2.279 [95% CI: 1.711–3.036; *p* < 0.001]). These findings indicate that prolonged decision‐making delay is a key independent determinant of prolonged door‐to‐CT time.

### 3.4 Two‐Stage Pooled Analysis Across Nine Hospitals

**FIGURE 3:**
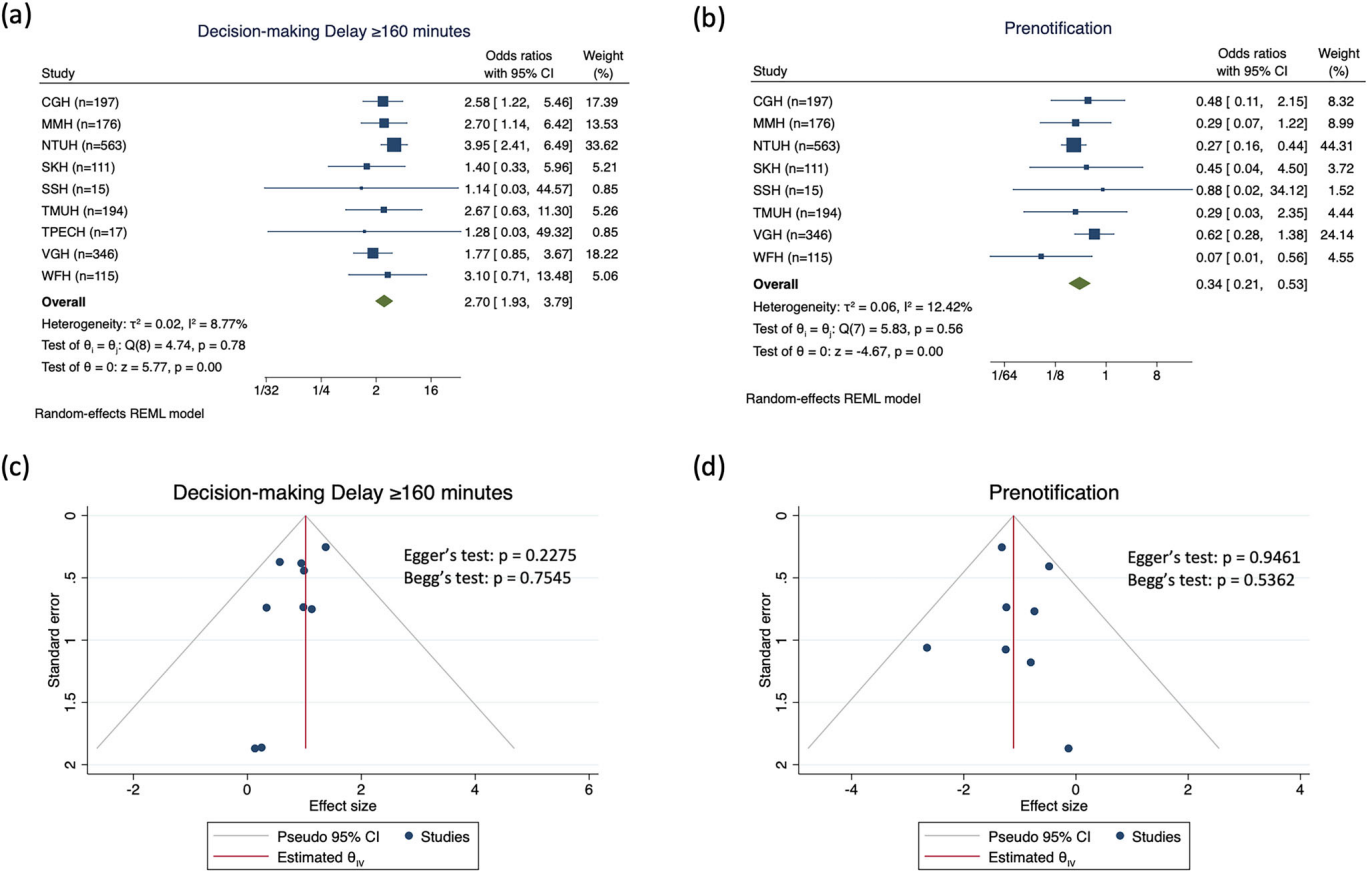
Two‐stage pooled analysis of the effects of decision‐making delay and prenotification on door‐to‐computed tomography (CT) time across nine advanced emergency responsibility hospitals. A DerSimonian‐Laird random‐effects model was used. (a) Forest plot showing the pooled odds ratio for decision‐making delays ≥ 160 min. (b) Forest plot showing the pooled odds ratio for prehospital notification. (c) Funnel plot assessing publication bias for decision‐making delays ≥ 160 min (Egger's test: *p* = 0.2275; Begg's test: *p* = 0.7545). (d) Funnel plot assessing publication bias for prehospital notification (Egger's test: *p* = 0.9461; Begg's test: *p* = 0.5362). CI, confidence interval; REML, restricted maximum likelihood.

The results of the two‐stage pooled analysis, including forest plots and funnel plots for the nine hospitals are presented in Figure 3. The Galbraith plots are shown in Figure S1. The pooled OR for a decision‐making delay of ≥160 min was 2.70 (95% CI: 1.93–3.79; *p* < 0.001; *I* ^2^ = 8.77%), indicating a significant association between decision‐making delay and delayed CT imaging as well as low heterogeneity across the hospitals. Similarly, the pooled OR for prenotification was 0.34 (95% CI: 0.21–0.53; *p* < 0.001; *I* ^2^ = 12.42%), indicating a strong protective effect of EMS prenotification on door‐to‐CT time as well as low heterogeneity across the hospitals. The funnel plots for both decision‐making delay ≥ 160 min and prenotification appeared symmetrical. Egger's and Begg's tests yielded no outlier hospitals. Moreover, the Galbraith plots revealed no outliers, confirming the robustness of the pooled analysis.

## 4 Discussion

This study demonstrated that decision‐making delay was significantly associated with prolonged door‐to‐CT time (>25 min). EMS prenotification reduced the time required for brain CT acquisition. A decision‐making delay of ≥160 min emerged as a key risk factor for prolonged door‐to‐CT time, underscoring the urgent need to enhance public awareness regarding the recognition of stroke symptoms and the importance of rapid response. Timely identification of the symptoms of acute ischemic stroke and immediate activation of the EMS are essential for optimizing clinical outcomes. Furthermore, EMS prenotification enables hospital medical teams to initiate prompt evaluation and intervention, reducing delays and improving care.

### 4.1 Decision‐Making Delay

Our findings are consistent with those of research highlighting the effect of decision‐making delays on door‐to‐CT time in acute ischemic stroke (Li et al. 2021). Zachrison et al. (2023) identified public recognition of stroke symptoms as a pivotal step in the chain of survival, highlighting the importance of early symptom identification and rapid EMS activation in minimizing prehospital delays and enhancing the timeliness and quality of stroke care. In our study, a decision‐making delay exceeding the average of 160 min was associated with a 2.3‐fold increase in the likelihood of prolonged door‐to‐CT times. Notably, extended decision‐making delays—particularly those surpassing the therapeutic window for IVT—may disqualify many patients from IVT eligibility and contribute to further in‐hospital delays in achieving definitive evaluation and management, including CT imaging (Evenson et al. 2009). Familial involvement greatly impacts stroke response times. García Ruiz et al. (2020) found that the presence of a family member, especially a son or daughter, often leads to quicker action. They emphasized the importance of responding immediately, regardless of symptom severity, and called for expanded public education to improve stroke awareness. These efforts should include a wider range of symptoms and target high‐risk groups such as those living alone, individuals with low incomes, and populations facing poverty and housing instability (Dhand et al. 2024; Fladt et al. 2019; Jain et al. 2022).

### 4.2 Prenotification

A significant association was noted between prehospital notification and reduced door‐to‐CT time. Prenotification enables receiving hospitals to prepare in advance for incoming patients with stroke, streamlining critical in‐hospital processes such as initial assessment, imaging, and laboratory testing (Abdullah et al. 2008; McKinney et al. 2013; Patel et al. 2011). A multicenter study conducted in Taipei demonstrated that prehospital notification substantially reduced door‐to‐CT time (Hsieh et al. 2016). Expanding on these findings, the present study, which had a long study period and a large patient cohort, substantiated the benefits of prenotification within Taipei City. At Taipei's advanced emergency responsibility hospitals, EMT‐initiated prenotification activates a standardized protocol designed to expedite patient transport and emergency care. Upon receiving prenotification, a designated entry point is prepared to facilitate the immediate transfer of patients with suspected stroke. This protocol allows these patients to bypass ED triage and proceed directly to the resuscitation area, where physicians can perform an expedited evaluation. Thus, brain CT imaging can be performed promptly, ensuring timely recanalization and IVT or IAT.

### 4.3 Hypertension

Hypertension was significantly associated with prolonged door‐to‐CT time in this study. A Korean study reported that hypertension predicted prehospital delays, with an OR of 1.12 (95% CI: 1.08–1.16) (Lee et al. 2024). By contrast, in the present study, the OR for a door‐to‐CT time of >25 min was 1.412 (95% CI: 1.047–1.905). A study conducted in the United Kingdom highlighted IV cannulation as a factor contributing to delayed ambulance response times (McClelland et al. 2023). Similarly, in Taiwan, IV cannulation is routinely performed upon arrival at the ED to facilitate drug administration; this potentially extends door‐to‐CT time. The AHA guidelines recommend maintaining blood pressure below 185/110 mmHg before the administration of tissue plasminogen activator (Powers et al. 2018). We hypothesize that for patients with a known history of hypertension, IV cannulation in anticipation of acute blood pressure management may inadvertently delay diagnostic imaging. However, because our dataset lacks initial blood pressure readings and specific records of acute therapies administered prior to imaging, this remains an exploratory observation. Although initiating blood pressure management before arrival at the ED may reduce delay, a 2024 clinical trial revealed no notable benefit from early blood pressure reduction in patients with undifferentiated stroke (Li et al. 2024). This finding underscores the inconsistency in the optimal protocol for prehospital management of hypertension in patients with acute stroke.

### 4.4 Clinical Implications

This study underscores the importance of public awareness in the early recognition of stroke symptoms. Timely identification and prompt action are essential for optimizing outcomes in acute ischemic stroke because decision‐making delays contribute to prolonged prehospital times. Public health education initiatives—delivered through media campaigns, community outreach, and school‐based programs—are essential for equipping individuals with the necessary knowledge to recognize stroke symptoms and promptly seek emergency care. Prehospital notification plays a pivotal role in reducing door‐to‐CT time. By facilitating early activation of stroke teams and enhancing the preparedness of the ED, prenotification ensures a smooth transition from prehospital care to in‐hospital care. This coordinated approach minimizes delays, ensures timely imaging, and increases the likelihood of successful revascularization. Ultimately, these strategies improve clinical outcomes and reduce the risks of stroke‐related morbidity and mortality.

### 4.5 Limitations

This study has several limitations. First, during the initial phases of the study, EMTs in Taipei were unfamiliar with the Acute Stroke Statistics System of Taipei City dataset, which resulted in incomplete documentation and patient exclusion. However, subsequent EMT training and improved collaboration with hospitals enhanced data quality and professionalism, with recent records indicating that the majority of patients now undergo CT within the recommended timeframe. Second, this study was conducted exclusively in metropolitan areas, which may limit the generalizability of the findings to rural settings, where stroke care infrastructures and access to emergency services may be limited. Future studies should explore these dynamics in rural contexts to provide a comprehensive understanding of stroke care across diverse healthcare settings.

## 5 Conclusion

Decision‐making delays and prehospital notification appear to be key determinants of door‐to‐CT time in the management of acute ischemic stroke. Enhancing public awareness regarding the symptoms of stroke and the urgency of rapid response and improving coordination between prehospital and in‐hospital care teams can markedly expedite stroke assessment and treatment. Furthermore, implementing targeted educational initiatives and optimizing emergency response protocols may help optimize stroke outcomes and prevent stroke‐related morbidity and mortality.

## Author Contributions

**Kuo‐En Chen**: conceptualization, data curation, formal analysis, Writing – original draft. **Po‐Chun Wang**: conceptualization, data curation, formal analysis, writing – original draft. **Sheng‐Feng Lin**: conceptualization, writing – original draft, methodology, writing – review and editing, resources, supervision. **Hui‐An Lin**: conceptualization, writing – original draft, methodology, writing – review and editing, data curation, resources.

## Funding

This study was supported by Taipei Medical University Hospital, Taiwan (grant no. 115TMU‐TMUH‐22) and the National Science and Technology Council, Taiwan (grant no. NSTC 115‐2314‐B‐038‐124). Both grants were awarded to Sheng‐Feng Lin.

## Ethics Statements

This study was conducted in accordance with the Declaration of Helsinki. Ethical approval was obtained from the Joint Institutional Review Board of Taipei Medical University, Taiwan (approval number: N202110064).

## Conflicts of Interest

The authors declare no conflicts of interest.

## Declaration of AI Usage

Generative AI and AI‐assisted technologies were not used in the preparation of this work.

## Supporting information




**Supplementary Material**: brb371656‐sup‐0001‐SuppMat.pdf

## Data Availability

The data that support the findings of this study are available from the Taipei City Fire Department. Restrictions apply to the availability of these data, which were used under license for this study. Data are available from the authors upon reasonable request, subject to approval by an institutional review board and permission from the Taipei City Fire Department.
