# Supplementary material for: Effect of Prehospital Time on Door‐to‐Computed Tomography Time in Acute Ischemic Stroke: A Multicenter Study
Source: Brain Behav. 2026 Aug 5;16(8):e71656. doi: 10.1002/brb3.71656 (PMC13439207; doi:10.1002/brb3.71656)
Supplement: Supplementary file 1 [file BRB3-16-e71656-s001.pdf]

**Supplemental Materials.**

**Supplemental Table 1.** List of the nine advanced emergency responsibility hospitals assessed in the present study.

**Supplemental Figure 1.** The Galbraith plot for outcomes of (a) decision-making delays ≥ 160 minutes, and (b) prenotification.

**Supplemental Table 1.** List of the nine advanced emergency responsibility hospitals assessed in the present study.

| **Advanced emergency responsibility hospitals in Taipei City** |
|---|
| Cathay General Hospital (CGH) |
| Mackay Memorial Hospital (MMH) |
| National Taiwan University Hospital (NTUH) |
| Shin Kong Wu Ho-Su Memorial Hospital (SKH) |
| Tri-Service General Hospital, Songshan Branch (SSH) |
| Taipei Medical University Hospital (TMUH) |
| Taipei City Hospital, Renai Branch (TPECH) |
| Taipei Veterans General Hospital (VGH) |
| Wanfang Hospital (WFH) |

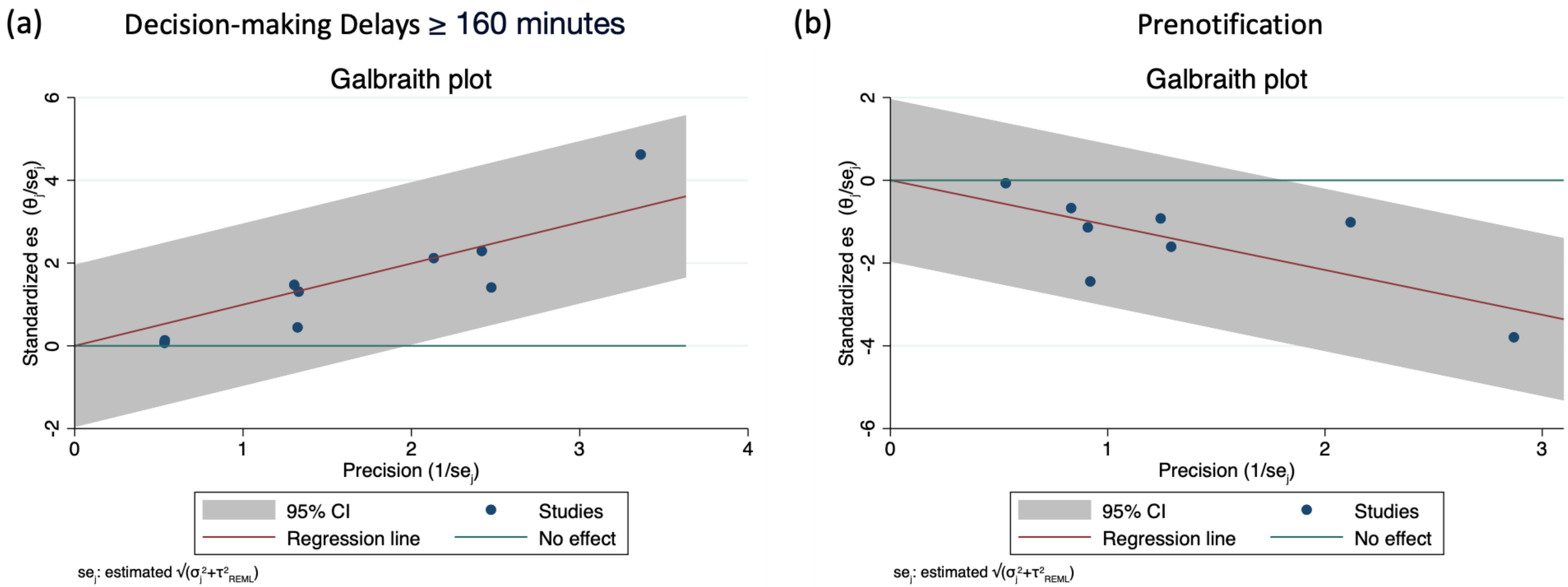


**Supplemental Figure 1.** The Galbraith plot for outcomes of (a) decision-making delays ≥ 160 minutes, and (b) prenotification.
